# Active Annotation in Evaluating the Credibility of Web-Based Medical Information: Guidelines for Creating Training Data Sets for Machine Learning

**DOI:** 10.2196/26065

**Published:** 2021-11-26

**Authors:** Aleksandra Nabożny, Bartłomiej Balcerzak, Adam Wierzbicki, Mikołaj Morzy, Małgorzata Chlabicz

**Affiliations:** 1 Department of Software Engineering Gdańsk University of Technology Gdańsk Poland; 2 Polish-Japanese Academy of Information Technology Warsaw Poland; 3 Faculty of Computing and Telecommunications Poznan University of Technology Poznań Poland; 4 Department of Population Medicine and Lifestyle Diseases Prevention Medical University of Białystok Białystok Poland

**Keywords:** active annotation, credibility, web-based medical information, fake news

## Abstract

**Background:**

The spread of false medical information on the web is rapidly accelerating. Establishing the credibility of web-based medical information has become a pressing necessity. Machine learning offers a solution that, when properly deployed, can be an effective tool in fighting medical misinformation on the web.

**Objective:**

The aim of this study is to present a comprehensive framework for designing and curating machine learning training data sets for web-based medical information credibility assessment. We show how to construct the annotation process. Our main objective is to support researchers from the medical and computer science communities. We offer guidelines on the preparation of data sets for machine learning models that can fight medical misinformation.

**Methods:**

We begin by providing the annotation protocol for medical experts involved in medical sentence credibility evaluation. The protocol is based on a qualitative study of our experimental data. To address the problem of insufficient initial labels, we propose a preprocessing pipeline for the batch of sentences to be assessed. It consists of representation learning, clustering, and reranking. We call this process active annotation.

**Results:**

We collected more than 10,000 annotations of statements related to selected medical subjects (psychiatry, cholesterol, autism, antibiotics, vaccines, steroids, birth methods, and food allergy testing) for less than US $7000 by employing 9 highly qualified annotators (certified medical professionals), and we release this data set to the general public. We developed an active annotation framework for more efficient annotation of noncredible medical statements. The application of qualitative analysis resulted in a better annotation protocol for our future efforts in data set creation.

**Conclusions:**

The results of the qualitative analysis support our claims of the efficacy of the presented method.

## Introduction

### Background

In 2020 and 2021, the world has not been fighting only a pandemic; more precisely, it has been fighting both a pandemic and an infodemic [1]. The spread of COVID-19 has been accompanied by an equally unfortunate and dangerous spread of misinformation such as fake news linking the COVID-19 epidemic to 5G technology [2]. Disinformation has influenced other disease outbreaks such as the measles outbreak in Germany that involved more than 570 reported measles cases and caused infant deaths [3]. This study suggests that there exist numerous similar examples. From anticholesterol treatment to psychiatry—potentially harmful noncredible medical content on varied topics proliferates on the web.

Web-based information related to health and medicine is a large and influential category of web content, to the extent that the term *Dr Google* has been coined. The case of health-related web content is interesting from the point of view of informatics not only because medical information is highly specialized and written using domain-specific vocabulary, but also because medical information on the web is often misinterpreted or taken out of context. Health-related fake news reports often rely on factually correct medical statements such as the antiseptic effect of silver ions, which translates into a false belief in the universal effectiveness of colloidal silver for treating any disease. Debunking health-related web content requires not only expertise but also awareness of the possible effects of misinterpreted information. The breadth of specialized medical knowledge, coupled with the impact of context on fake news debunking, increases the difficulty of the problem of medical fake news detection.

Fully automated methods are currently not mature enough to detect medical fake news with sufficient accuracy. A realistic system for detecting and debunking medical fake news needs to keep medical experts in the loop. However, such an approach is not scalable because medical experts and health professionals cannot allocate sufficient time to handle the volume of misinformation spreading on the web. Another issue is that, in general, compared with credible medical content, noncredible medical web content is sparse. Assuming a real human–assisted system for assessing the credibility of medical statements, statistically, out of 100 assessed statements, the expert will catch no more than 20 unreliable items (as shown by our data collection experiment). The purpose of our work is to create an automatic tool to maximize the number of potentially noncredible sentences to be verified in the first place. The sentences are then reordered so that the most noncredible content shows up first to be annotated by a human judge. In such a way, we can optimize medical experts’ time and efficacy when annotating medical information. Even if only a portion of potentially noncredible sentences gets annotated by the expert, it will include the most suspicious content.

We propose to use a method called active annotation. It dramatically improves the use of annotators’ time. Active annotation implements a highly efficient human-in-the-loop component for augmented text annotation. The main idea behind active annotation is to use an unsupervised machine learning method (grouping of sentences into clusters based on sentence similarity) to organize the training data to suggest annotation labels for human annotators. When active annotation is used, the work of human annotators (medical experts) is focused on difficult noncredible medical statements. In addition, because the annotators process clusters of semantically similar sentences, our method significantly reduces the cost of cognitively expensive context switching. However, it is the annotators who decide the final labeling of the data.

The method proposed in this paper extends currently known active annotation methods by a cluster-ranking procedure that ensures that medical experts first see the content clusters that are most likely to contain noncredible content. This approach allows us to speed up the discovery of noncredible content. In our view, the process of detecting and debunking medical misinformation will never stop, and therefore a method that optimizes the use of medical experts’ is of essential importance.

To test our method, we conducted an experiment with the participation of medical experts. They were asked to evaluate the credibility of medical and health-related Web content. The result of the experiment is a large data set that contains numerous examples of medical misinformation. We conducted an explorative and qualitative analysis of this data set, searching for patterns of similarity among the different examples of medical misinformation. The result of this analysis (which included an in-depth case study of misinformation related to cholesterol therapy with statins) was the discovery of distinct narratives of medical misinformation. We believe that these narratives are general in nature and will be of great use for detecting medical misinformation in the future.

Our direct experiences with the annotation team dictate a set of rules that have been formalized as a strict protocol for medical text annotation. Most importantly, we noted that the annotators tended to use external contexts extensively when annotating data. This, in turn, led to incoherent annotation labels across the data set and a divergence between the notions of statement credibility and statement truthfulness. We share our experience and present an annotation protocol that we have used to mitigate some of the annotation problems.

The original contributions presented in our paper are as follows:

- An annotation schema, an annotation protocol, and a unique annotated data set comprising 10,000 sentences taken from web-based content on medical issues, labeled by medical experts as credible, noncredible, or neutral. The entire data set is available in a public repository [4].
- A method for ranking sentences submitted to medical experts for labeling. Our active annotation method increases the likelihood that medical experts will discover noncredible sentences and thus optimizes the use of medical experts’ time.
- A qualitative analysis of the labeled data set. We discovered 4 distinct narratives (both syntactic and semantic) present in the noncredible statements. We believe that these narratives can be further used to discern noncredible statements in areas of medicine other than the areas covered by our data set.

### Literature Review

Health literacy is a rising concern, especially during the COVID-19 pandemic. However, research shows that more than half of the population struggles with making proper judgments and taking decisions in everyday life concerning their health [5]. Moreover, studies from the United States, Europe, and Australia [6,7] have found that web-based health information is written above the average reading level of adults. There is clearly the need for external tools or strategies to support laypersons in assessing the credibility of web-based health information. Expert fact-checking is one of the proposed strategies [8] because short-format refutational medical expert fact-checks have proven to be free from the *backfire effect* [9] (the *backfire effect* has been described in the study by Nyhan and Reifler [10]). Research shows that using expert sources to correct health misinformation in social media permanently corrects users’ false beliefs.

The related work on the general news media domain [11] demonstrates that a credible source can promote false information and vice versa. Technological innovation in the fight against disinformation, as the authors argue, should go beyond discrediting noncredible sources of information and should instead promote more careful information consumption [11]. The literature has reported on successful machine learning models that classify entire articles or information sources [12,13]. Of note, these models can easily overfit (ie, obtain high classification accuracy for publications from media outlets present in the training set but fail to generalize to previously unseen media outlets). The possible performance drop in classifying fake news from previously unseen sources has been examined in the literature [12]. The study by Afsana et al [14] is, to the best of our knowledge, the most accurate classification model for assessing the quality of web-based health information. The authors declare accuracy ranging from 84% to 90% varied over 10 criteria. The model includes source-level and article-level features. The relationship of the described criteria with credibility remains an open research question.

The assessment of the veracity of individual claims contained in open-domain news articles is an emerging and fast-growing field of research. The scope of activities includes the creation of data sets containing the claims collected from fact-checking websites, such as MultiFC [15], Liar [16], and Truth of Varying Shades [17], and the existing solutions are based on a variety of approaches, from semi-automatic knowledge graph creation [18] to choosing check-worthy claims and comparing them against verified content (ClaimBuster) [19]. The open-domain solutions or solutions used in journalism [20] are not easily transferable to the medical domain.

The MedFact system [21] is a stand-alone web-based health information consumption support system. In MedFact, the user is automatically equipped with relevant trusted sources during web-based discussions.

State-of-the-art information retrieval models [22,23] forms part of the fully and semi-automatic fact-checking systems. A combination of such systems’ judgments and human judgments has been successfully applied in the study by Ghenai and Mejova [24] for the specific case of capturing the spread of rumors regarding the Zika virus. Our goal is to test the combination of an unsupervised machine learning model with a human-in-the-loop approach as a robust tool to support the assessment of the credibility of web-based medical statements.

The quality assessment coding scheme for lay medical articles had been proposed in the 1990s under the Discern handbook project [25] and as Health on the Net (HON) principles. However, the guidelines have to comply with the rapidly evolving web-based reality; thus, new tools and updates are designed every few years, such as the Ensuring Quality Information for Patients (2004) [26] tool, Evidence-Based Patient Information (2010) [27], and Good Practice Guidelines for Health Information (2016) [28], to name a few. Keselman et al [29] propose different credibility assessment criteria based on 25 web-based articles regarding type 2 diabetes. These criteria (objectivity, emotional appeal, promises, and certainty) can be automatically captured by language models and lexicon-based machine learning. Work on web-based journalism has developed good practices that can also be used by medical experts in credibility evaluation. Medical practitioners who directly communicate medical information to patients can observe their reactions and subsequent actions and therefore have a special agency in credibility evaluation.

Successful application of machine learning models requires the annotation of vast corpora of medical information. However, this annotation is prohibitively expensive given the required expertise of the annotators and their limited capacity. Active annotation is a technique that facilitates large-scale data annotation by providing an auxiliary ranking of sentences that should be manually annotated by medical experts and by expediating labeling of other sentences to the underlying machine learning model. In this study, we are particularly inspired by the approach presented by Marinelli et al [30]. The authors propose initially dividing text documents into separate clusters, selecting pivot documents (k-closest documents to the center of each cluster), and generating a tentative label for the cluster. Next, a small set of text documents is selected and presented to human annotators with a proposed label and a binary annotation decision (to accept or reject the label). The authors claim that in many applications, obtaining a full annotation schema before annotation may be difficult and turning the annotation task into a binary question–answering task significantly speeds up the process [30].

### Language Modeling

The term *language model* is confusing because it serves as an umbrella term for different concepts. As a general rule, a language model is a way in which textual content (tokens, words, sentences, paragraphs, and documents) is represented. Historically, text documents have been represented using 2 prevalent models: the bag-of-words model (where a document is represented simply as the set of words appearing in the document) and the one-hot encoding model (where a document is represented by a binary vector of a length equal to the size of the vocabulary and each position in the vector encodes the presence or absence of a word in the document). The most consequential limitation of these models was the inability to capture the semantic similarity between words. For instance, if a document contained the word *diabetes* and another document contained the word *insulin*, there was no straightforward way of deciding that the documents shared a common topic. This limitation has been abruptly neutralized with the advent of word embeddings. Word embeddings are dense continuous vector representations of words from a given vocabulary, which means that each word is assigned a unique vector whose elements are arbitrary numbers. Unlike one-hot encoding vectors where each vector has a length equal to the size of the vocabulary, word embedding vectors have, at most, several hundred dimensions. The vectors are trained on the text corpus to capture various semantic relationships among words. For instance, words such as *apple*, *pear,* and *orange* appear close to each other in the vector space because part of their representation encodes the notion of being a fruit. Analogically, the distance between the words *Russia* and *Moscow* is similar to the distance between the words *Great Britain* and *London* because the difference between the respective word vectors encodes the notion of a capital city.

Since the seminal work of Mikolov et al [31], word embeddings have revolutionized the field of natural language processing. After the initial success of the *word2vec* algorithm, numerous alternatives have been introduced: Global Vector embeddings trained through matrix factorization [32], embeddings trained on sentence dependency parse trees [33], embeddings in the hyperbolic space [34], subword embeddings [35], and many more. The common feature of these embeddings is the static assignment of dense vector representations to words. Each word receives the same embedding vector, irrespective of the context in which the word appears in a sentence. These static embeddings can be used to create representations for larger text units such as sentences, paragraphs, and documents. However, static embeddings are inherently unable to capture the intricacies hidden in the structure of the language and encoded in the context in which each word appears. Consider these 2 sentences: “A photo reveals significant damage to the tissue” and “Please do not throw used tissues into the toilet.” The word *tissue* will receive the same vector although the context allows disambiguation of the meaning of the word.

To mitigate this limitation, modern language models depend on deep neural network architectures to calculate accurate, context-dependent word and sentence embeddings. First, context-dependent language models used either the long short-term memory network architecture [36] or gated recurrent unit networks [37] to capture contextual dependencies among the words appearing in a sentence. In other words, unlike static word embeddings, context-dependent language models calculate an embedding word vector based on the context (ie, words surrounding the embedded words). In the aforementioned example, the word *tissue* would receive 2 different vector representations: in the first sentence, the vector for the word *tissue* would be much closer to the vectors of words such as *skin* or *cell*; in the second sentence, the vector for the word *tissue* would be closer to the vector of the word *handkerchief*. These early recurrent architectures, however, suffered from performance drawbacks, and in 2018 they were replaced by transformer architecture [38]. This architecture allowed the training of much better embeddings, such as Google’s Universal Sentence Encoder [39] or the (infamous) Generative Pre-trained Transformer 3 [40].

The current state-of-the-art language model, Bidirectional Encoder Representations from Transformers (BERT) [41], produces continuous word vector representations by training the neural network using 2 parallel objectives: guessing the masked word in a sentence (ie, trying to predict the word based on the context) and deciding whether 2 sentences appear one after another. Given such training objectives, the network applies similar weights to the nodes regarding input words that appear in a similar context. Sentence-BERT (sBERT) [42] is a straightforward extension of the original BERT architecture for creating sentence embeddings. This model is based on Siamese BERT networks [43] (2 identical models trained simultaneously) that are fine-tuned on the Natural Language Inference and Semantic Textual Similarity tasks. The model serves as an encoder for sentences. The encoder calculates vector representations of sentences so that semantically similar sentences have low cosine distance in the latent embedding space. This is both more efficient and produces semantically richer sentence representations than simply averaging the vectors of words that appear in each sentence.

## Methods

### Presentation of 3 Steps

To validate the efficacy of the active annotation approach, we need to create a data set of sentences on medical topics gathered from the Web, after which we need to obtain credibility evaluations of these sentences from medical experts. We need to propose methods for selecting sentences from the Web, annotating of these sentences by medical experts, and organizing these sentences into a processing pipeline to use the experts’ time and attention most efficiently. These 3 steps we elaborate on in this section.

### Data Selection

We performed annotation on a data set of 247 articles collected manually from various eHealth websites. The data set consists of more than 10,000 sentences. All documents were annotated by medical professionals sentence by sentence. The sentences constitute a stratified sample of source texts of varying credibility. We first discussed the most problematic topics of specific medical fields with the medical practitioners. Next, we manually searched for articles that presented contradicting views regarding these topics. These topics include the following:

1. Pediatrics:
  Children’s antibiotics consumption (432 sentences)
  Children’s steroids consumption (701 sentences)
  Vaccination (1262 sentences)
  Dietary interventions for children with autism (431 sentences)
  Food allergy testing (1401 sentences)
2. Psychiatry:
  Effectiveness of psychiatric medication and electroconvulsive therapy (2272 sentences)
3. Cardiology:
  Benefits of statin therapy in treating cardiovascular disease (CVD; 2029 sentences)
  Dietary interventions for heart health improvement (423 sentences)
  Benefits of consumption of antioxidants (694 sentences)
4. Gynecology:
  Benefits of cesarean section over natural birth (359 sentences)
  Selective serotonin reuptake inhibitor consumption during pregnancy (169 sentences)
  Aspirin consumption during pregnancy (257 sentences)

Our collection of web-based health-related and medical articles reflects topics potentially causing controversy and misinformation among patients.

### Methodology of Selecting Source Websites

The source websites were selected as follows. First, we asked each medical practitioner 2 questions:

1. “In your medical practice, what kind of false beliefs and rumors do you encounter when interacting with patients?”
2. “The truthfulness of which facts do you have to prove to your patients most often?”

The answers to these questions served as the basis for manually creating web queries. To create a data set of web medical articles addressed to laypersons, we submitted these queries to the Google search engine and then manually selected sources. The full list of these queries is listed in Multimedia Appendix 1. The manual collection was supported by the HON browser plugin (HON tag–certified webpages). As a result, 12.6% (31/247) of the extracted articles originated from HON-certified sources. The remaining 87.4% (216/247) come from domains such as the following:

- Large news media outlets (eg, *The Guardian*, *The New York Times*, and BBC)
- Q&A forums, both general and topic-specific (eg, “Quora”, “Yahoo”, “community.babycenter.com”)
- Parenting blogs (eg, “scarymommy.com”)
- Uncertified health portals (eg, “choosingwisely.org”, “practo.com”, and “heartuk.org.uk”)
- Advertising websites for medical supplements and medical testing (eg, “everlywell.com”, “yorktest.com”, and “naturesbest.co.uk/antioxidants”)

The full list of data sources is available in Multimedia Appendix 2.

In this study, we consider a sentence as the unit of consistent information that undergoes credibility assessment. According to Wikipedia [44], “a sentence is a set of words that in principle tells a complete thought.” Thus, unless a sentence is highly complex, we can assume that the segmentation of a document into sentences is the easiest way to automatically extract single statements. To be precise, a single sentence may contain several statements. We have also observed that expert annotators tend to focus on statements rather than entire sentences when labeling data. However, we do not have a robust method of statement demarcation. In addition, most sentences contain a single main statement; thus, we decided to make the sentence the atomic unit of annotation and classification.

An additional reason for focusing on single sentences is the phenomenon of shrinking attention. Recent studies suggest that, over recent decades, collective attention spans are becoming shorter across all domains of culture, including the web [45]. It is debatable as to what the underlying cause of this phenomenon is. The most likely explanations suggest the impact of the rapid acceleration in the rate of production and consumption of information. Given finite attention resources, this inevitably leads to more cursory interaction with information. It is possible that this phenomenon also affects the consumption of health-related information, which only exacerbates the problem of the ubiquitousness of medical fake news on the web.

### Expert Annotators

In all, 9 medical professionals took part in the experiment: 2 cardiologists, 1 gynecologist, 3 psychiatrists, and 3 pediatricians. All the experts had completed 6 years of medical studies, followed by a 5-year specialization program that culminated in a specialization examination. The experts were paid for a full day of work (approximately 8 hours each). Of the 9 experts, 8 (89%) had at least 10 years of clinical experience. The gynecologist was a resident physician; we accepted his participation in the experiment because of his status as a PhD candidate in medicine. Of the 3 psychiatrists, 1 (33%) held a PhD degree in medical sciences. The experts were allowed to browse certified medical information databases throughout the experiment. Each expert evaluated the credibility of content within their specialization (cardiology, gynecology, psychiatry, or pediatrics).

### Annotation Protocol

Our goal is to create a rich and diverse corpus of medical sentences assessed and labeled in terms of their credibility by medical experts. To obtain reliable and comparable credibility evaluations, the experts participating in our study were supported by a detailed annotation protocol.

The medical experts evaluated the credibility of sentences with the following set of labels and the corresponding instruction:

- CRED (credible): the sentence is reliable; does not raise major objections; contains verifiable information from the medical domain
- NONCRED (not credible): the sentence contains false or unverifiable information; contains persuasion contrary to current medical recommendations; contains outdated information
- NEU (neutral): the sentence does not contain factual information (eg, it is a question); is not related to medicine

The experts were asked to base their answers mostly on their experience, knowledge, and intuition, but they were also allowed to use an external database that they would usually use in the course of their medical practice. The main direction provided to the experts was to focus on the patient’s alleged perception of the information. The control question stated as follows: “If the patient asked you if he or she should trust this statement, would you say yes or no?”

In addition, we collected the following information for each sentence:

- Time needed for evaluation (in milliseconds)
- (Optional) Reason for evaluating the sentence as noncredible
- Number of surrounding sentences needed to understand the context of the sentence being evaluated

Examples of credible sentences from the *cholesterol and statins* topic include the following:

Lp(a), the worst cholesterol, is a number most doctors don’t measure.

Monitoring cholesterol levels is crucial because individuals with unhealthy cholesterol levels typically do not develop specific symptoms.

Non-communicable chronic disease is now the biggest killer on the planet.

Examples of noncredible sentences include the following:

For the remaining 90% of the population, the total cholesterol had no predictive value.

It seems likely that fear of fat is unreal, based on a carry-on of the cholesterol fear.

Most people don’t need to cut down on the cholesterol that’s found in these foods.

Examples of neutral sentences include the following:

Seven [research items] found no link between LDL cholesterol and cardiovascular mortality.

These perspectives won’t make headlines and they won’t appeal to those who want a simple and definite answers.

This is not why I went to medical school.

### Impact of Sentence Context on Credibility Evaluation

Table 1 shows how many sentences required additional *m*-surrounding sentences to provide the context for annotation. When focusing on noncredible statements, more than 71.27% (1377/1932) of the sentences were self-explanatory, 26.6% (514/1932) of the sentences required a single sentence of context, and less than 2.17% (42/1932) of the sentences required 2 or more sentences of context. Thus, we conclude that our choice of the sentence as the unit of information is justified.

**Table 1:** Number of surrounding sentences (*m*) needed to understand the context and evaluate the credibility of a sentence for all data, only credible subset, only noncredible subset, and only neutral subset (n=10,649).

*m*	All data, n (%)	Credible subset, n (%)	Noncredible subset, n (%)	Neutral subset, n (%)
0	8565 (80.43)	4955 (80.07)	1377 (71.27)	2233 (88.3)
1	1958 (18.39)	1165 (18.83)	514 (26.6)	279 (11.03)
2	107 (1)	57 (0.92)	34 (1.76)	16 (0.63)
3	12 (0.11)	5 (0.08)	6 (0.31)	1 (0.04)
<3	8 (0.07)	6 (0.1)	2 (0.05)	0 (0)

**Figure 1:**
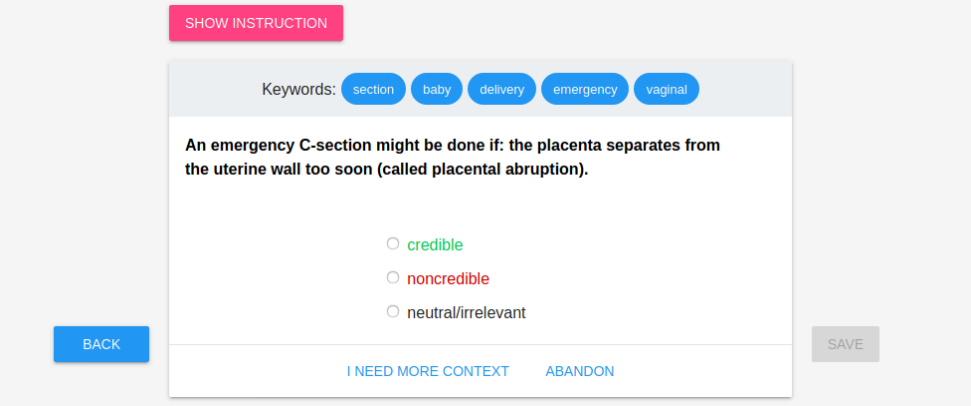
Annotation interface: single sentence view.

For the annotation process, we used the software developed specifically for this experiment. During the experiment, the medical expert could not see the context of the whole document while annotating a sentence. However, we provided the most relevant keywords collected from the rest of the document. Keywords were extracted using the methods described in the study by Nabożny et al [46]. A single task is shown in Figure 1.

**Figure 2:**
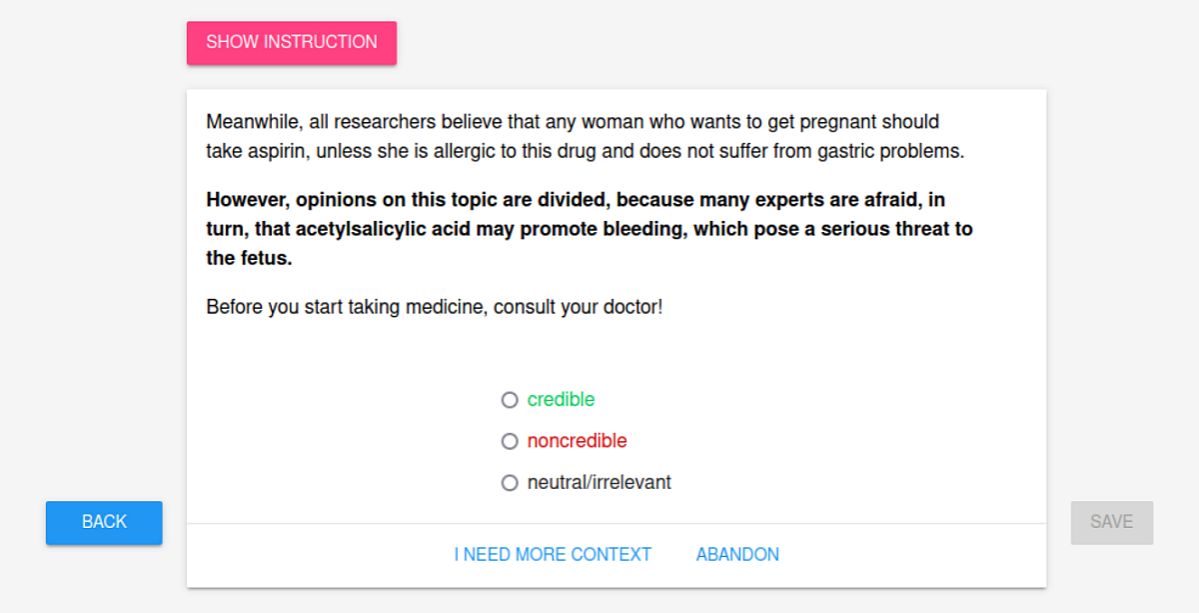
Annotation interface: sentence in context view.

If the medical expert decided that a sentence could not be assessed because of insufficient context (despite visible keywords), they could display the preceding and succeeding sentences in the annotation view, as shown in Figure 2. Each medical expert was asked to annotate approximately 1000 randomly chosen sentences. Whenever the medical expert labeled a sentence as noncredible, they were asked to provide the reason for their decision. To avoid the effect of intentionally skipping the NONCRED label to complete the task quicker, providing the reason was optional, and the expert could also choose an explanation from a set of tags prepared beforehand.

The set of possible explanations prepared in advance included the following:

- The sentence contains argumentation that is weak or irrelevant, given the context of the subject being discussed.
- The sentence contains an encouragement to act inconsistently with current medical knowledge.
- The author of this sentence shows signs of the lack of substantive knowledge or is not objective.
- The sentence is an anecdote or a rumor.
- The sentence is an advertisement of an unproven drug or substance or an unproven therapy.
- The sentence cites research that was conducted on a small sample.
- The sentence contains invalid numerical data.
- The sentence contains outdated information.
- The sentence is incomprehensible or grammatically incorrect.

Most of the annotation was conducted in controlled laboratory conditions. The experts were performing annotation tasks in the presence of a supervisor who was conducting the experiment. At any time, the medical experts had access to the detailed instruction (definitions of each label) and could also ask the supervisor for assistance. The experts completed 70% of the tasks in controlled conditions, and the rest were completed with web-based assistance within a few days after the conclusion of the laboratory experiment.

### Sentence Processing Pipeline Using Clustering and Reranking

Inspired by the active learning paradigm, we designed an assessment loop for medical sentence credibility. The core idea of the active annotation approach is to augment annotation efforts by 2 mechanisms:

- *Clustering*:
  Semantically similar sentences are automatically grouped into clusters. The process of clustering uses sentence-embedding representation. Each sentence is represented as a vector computed by the language model. As each sentence is a vector, mathematical measures of a distance can be used, such as the Euclidean distance or the cosine distance. We use the k-means algorithm to divide sentences into clusters. K-means is a simple iterative procedure where clustered items (in our case, vectors representing sentences) are assigned to the closest of k points representing cluster centers (also known as centroids). After assigning each item to the nearest centroid, the positions of the centroids are updated to reflect the geometric mean of assigned items. Finally, items are reassigned to the nearest centroid, and the procedure is repeated until no more reassignments are possible. The resulting clustering maximizes the similarity among the items assigned to a cluster and at the same time minimizes the similarity among the items assigned to different clusters. In other words, if 2 sentences are assigned to the same cluster, the distance between their vector representations is small, which in turn means that the sentences are semantically similar (because semantic similarity is the criterion of embedding vector training). When human annotators are presented with sentences from a cluster, they process sentences that share a common topic. This reduces the cognitive workload of human annotators because they do not have to switch contexts between annotated sentences.
- *Reranking*:
  Noncredible statements are moved to the top of the ranking. Human annotators are required to identify noncredible statements; thus, every time human annotators are presented with a credible or neutral sentence, they may consider it to be a waste of their precious time. By combining sentence embeddings and clustering, we push sentences that are close to the already labeled noncredible sentences to the top of the ranking, prioritizing these sentences for the next round of manual annotation.

In the active annotation process, the following steps are performed in the assessment loop:

1. Sentences from the corpus are encoded by the language model to produce sentence embeddings.
2. The k-means clustering algorithm [47] is applied, and the top *k* sentences nearest to the cluster center are chosen for initial human annotation. We use the elbow method [48] to find the number of clusters (which represents the number of distinct topics in the corpus).
3. Medical experts annotate selected sentences.
4. The algorithm reranks all sentences based on the distribution of labels within clusters.
5. Medical experts annotate sentences from the top of the ranking, triggering another reranking procedure.

**Figure 3:**
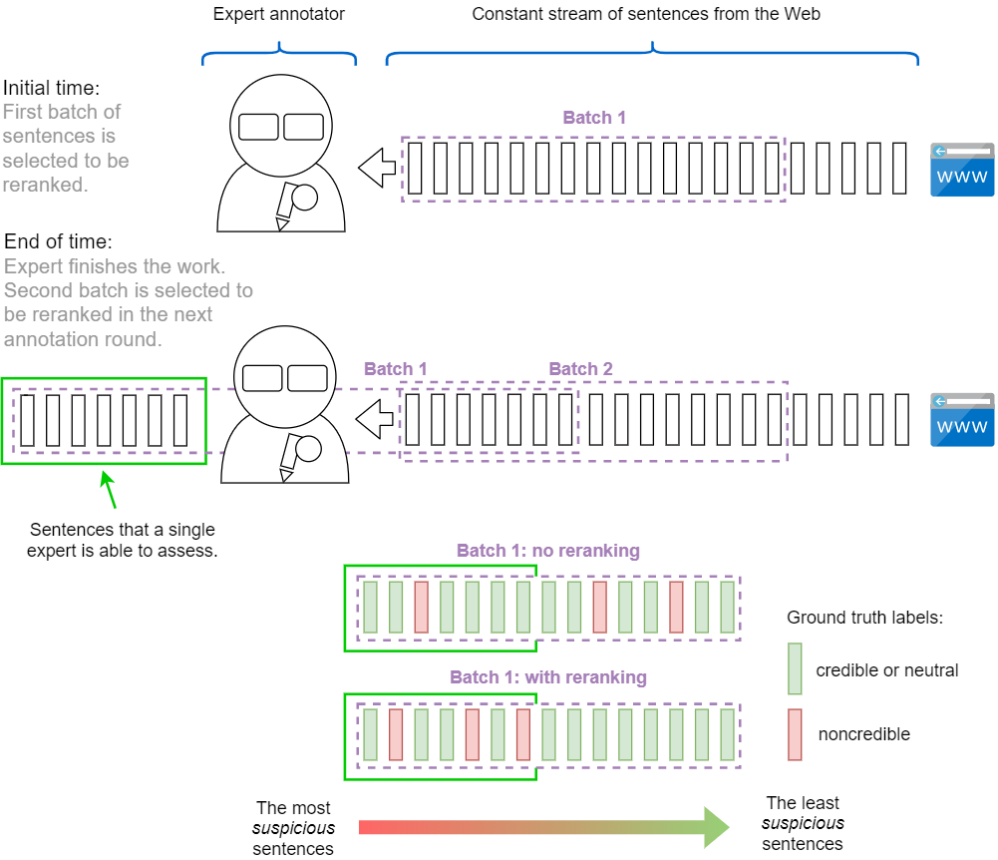
Sentence reranking: general idea.

The general idea behind reranking is presented in Figure 3.

Step 4 is crucial to the method. First, we find clusters with a large proportion of labeled noncredible statements. During initial iterations of the method, only a small fraction of sentences are manually labeled, but the clustering step groups semantically similar sentences; therefore, we expect that many sentences belonging to a cluster with predominantly noncredible labels also would turn out to be noncredible. In step 5, more sentences are manually labeled, providing a better approximation of the true distribution of labels within clusters. By repeating steps 4 and 5, we annotate more and more sentences, prioritizing the annotation of noncredible sentences.

For sentence embeddings computations, we use the sBERT modification Robustly Optimized BERT Pretraining Approach where embeddings are calculated based on the same model as BERT but with slightly different training objectives and hyperparameters [49]. We also use a simple preprocessing technique where we subtract the mean and exclude the first principal component from each embedding vector [50,51] (principal component analysis transformation). The assumption behind this step is that the first principal component encodes syntactic rules of the grammar of the sentences without contributing to their semantics. The removal of the first component strips sentence vectors of grammar and leaves only the part of the vector where the meaning is encoded.

**Figure 4:**
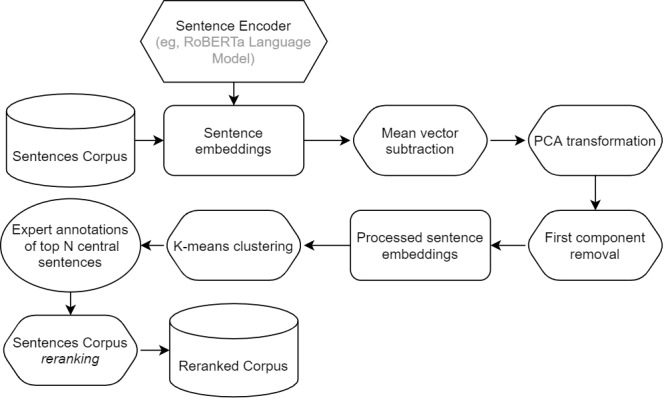
Processing pipeline. PCA: principal component analysis; RoBERTa: Robustly Optimized Bidirectional Encoder Representations from Transformers Pretraining Approach.

Figure 4 presents the overview of the sentence processing pipeline.

The key component of the pipeline is the clustering and reranking strategy. For reranking, we perform 2-level sorting. The first sorting is applied to clusters, and the second sorting reorders sentences within clusters. We rank clusters based on the proportions of credible, noncredible, and neutral labels in the top *m* most central sentences. Our scoring formula penalizes clusters with a significant proportion of credible sentences. At the same time, it rewards clusters with a significant proportion of noncredible sentences. This strategy enables us to push most of the noncredible sentences to the top of the ranking, thus positioning them at the top of the queue for medical expert evaluation.

Let p(c), p(n), and p(u) denote the probability that a random sentence is credible, noncredible, or neutral, respectively. This probability is computed by manually annotating *m* most central sentences in the cluster. The cluster score is defined as follows:

score@k = 1/e^–(p[n]–p[c])^ + 1/w^p(u)+1^ **(1)**

The first component of the formula is the sigmoid function with the difference between p(n) and p(c) as the argument. If the difference is positive, which means that there is an advantage of noncredible proportion over credible, the sigmoid function gives results close to 1 (the bigger the difference, the closer to 1). If the difference is negative, the sigmoid value tends toward zero. The second component of the formula is the parametrizable function, which enables giving proper scoring weight to p(u). For example, given w=1.5, it orders clusters with p(n)=0.4 and p(c)=0.3 below clusters with p(n)=0.5 and p(c)=0.4. Without the second component, both clusters would receive the same score.

The intracluster ranking of sentences is performed based on the distance of sentences from the center of the cluster, with more central sentences placed at the top of the ranking. The distance is measured as the cosine distance in the latent embedding space. The final ranking of all sentences is obtained by first ordering all clusters in the decreasing order of score@k and, next, by reordering sentences within each cluster by the growing distance from the center of the cluster.

## Results

### Overview

We used the method described in the previous section to create an annotated data set. We now describe the results. First, we present the data set statistics. Next, we depict the effect of our sentence pipelining method on the effectiveness of the medical experts’ time allocation. Subsequently, we conduct a qualitative analysis of the credible and noncredible sentences, focusing on a single topic.

### Distribution of Labels Within the Data Set

**Figure 5:**
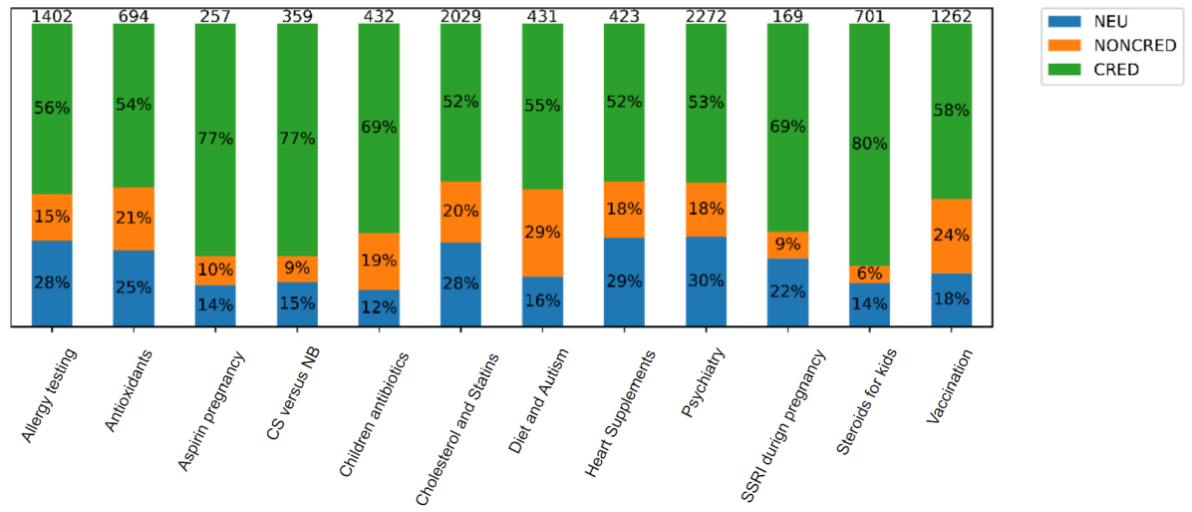
Distribution of credible, noncredible, and neutral sentence labels within topics. CS: cesarean section; CRED: credible; NB: natural birth; NEU: neutral; NONCRED: noncredible; SSRI: selective serotonin reuptake inhibitor.

The distribution of labels (CRED, NONCRED, and NEU) for each topic is shown in Figure 5. Distribution varies for each topic but within a certain range. For example, the CRED label is always at least two times more frequent than the NONCRED label and significantly more frequent than the NEU label. The NEU label applies to no more than 30% (3195/10,649) of the sentences in all topics, which leads us to the conclusion that, regardless of the topic, more than 59.99% (6389/10,649) of the statements warrant credibility checking.

Although the articles were explicitly picked so that they reflect potentially controversial topics, the proportion of noncredible sentences was generally small. Taking into account the alarm-raising calls of the medical experts, we can conclude that even a small contribution of noncredible content throughout the web has a substantial influence on the formation of people’s views.

### Justification for Using the Lift Measure

We have chosen the lift measure to evaluate the effectiveness of our method. Throughout the qualitative analysis, it became apparent that semantic similarity measures retrieved from neural language models lose important information encoded in annotations. Our objective is to optimize medical experts’ time by focusing their attention on statements that are possibly noncredible. Using the lift measure, we determined the relative time savings by indicating how many more noncredible sentences a medical expert would see by reviewing a given percentage of the entire sentence corpus using our ranking. The lift measure specified for each ranking percentile is defined as follows:

*lift@p* = N/p × *recall@p* **(2)**

where *p* is the percentile, *N* is the total number of sentences in the corpus, and *recall@p* defines, for a given percentile *p* of the ranking, how many noncredible statements have been included in the *p*th percentile of the ranking.

The key parameter of our method is *m*, the number of top sentences in a cluster for manual annotation. We tested our method on a full data set (all topics merged) for 3 *m* values*,* each of which is listed in Table 2. In Table 3, we present the lift results for the separate topic of *cholesterol and statins*. The baseline value for lift is 1. Thus, we can interpret the results as follows: the number by which a given value exceeds 1 tells us how many more noncredible sentences medical experts would discover at a given corpus percentile when using the reranking procedure. For example, when reviewing 20% of the full corpus, medical experts would discover 29% more noncredible sentences if the batch were to be reranked using the *m* value of 5 than without applying the procedure.

**Table 2:** Lift results for the full data set. *m* is the number of top sentences from each cluster to be manually reviewed.

lift@*m*	Number of clusters	Batch percentile			
		1% (approximately 100 sentences)	10% (approximately 1000 sentences)	20%	40%
lift@5	200	1.36	*1.36* ^a^	*1.29*	*1.17*
lift@10	130	1.23	1.31	1.3	*1.17*
lift@15	100	*1.49*	1.27	1.22	1.16

^a^The best performing set of parameters for a given batch percentile is italicized.

**Table 3:** Lift results for the cholesterol and statins topic. *m* is the number of top sentences from each cluster to be manually reviewed.

lift@*m*	Number of clusters	Batch percentile			
		1% (approximately 20 sentences)	10% (approximately 200 sentences)	20%	40%
lift@5	40	1.75	1.24	1.26	1.27

The number of clusters for each experiment is chosen based on 2 criteria: the elbow method [48] and the proportion of sentences to be manually reviewed. The latter should not exceed 15% of the batch. Let us take Table 3 as an example: we delegate 5 × 40 = 200 top sentences from each cluster to be manually reviewed by the experts. These 200 sentences out of the approximately 2000 sentences in the *cholesterol and statins* topical category make up 10% of the set. It means that by gathering initial labels from only 10% of the sentences from the topical corpus, we can obtain significant (eg, 27% in the 40th percentile) savings of experts’ time during text annotation sessions.

### Zooming in on a Topical Cluster: Case Study of Statins

We conducted a case study in the subdomain of cholesterol and statins. We did this to gain insight into the process of credibility evaluation and the nature of noncredible medical sentences. The focus on a single topic was dictated by the size and diversity of our data set. Presenting an in-depth qualitative analysis of the entire data set would take too much space. The following is a qualitative analysis of all sentences labeled noncredible by the experts in the selected topic.

### Brief Introduction to the Topic of Statin Use

Numerous epidemiological studies, Mendelian randomization studies, and randomized controlled trials have consistently demonstrated a relationship between the absolute changes in plasma low-density lipoprotein (LDL) and the risk of atheromatous CVD. The inverse association between plasma high-density lipoprotein and the risk of CVD is among the most consistent and reproducible associations in observational epidemiology. Higher plasma Lp(a) concentrations are associated with an increased risk of CVD, but it appears to be a much weaker risk factor for most people than LDL cholesterol [52]. Commonly, plasma cholesterol is used to calculate cardiovascular risk, whereas LDL is used to evaluate the achieving of target values according to the estimated cardiovascular risk.

Hypercholesterolemia (dyslipidemia with an increased levels of circulating cholesterol) is not the only factor responsible for the development of CVD, but also obesity, poor diet, lack of physical activity, smoking, and high blood pressure (hypertension). To prevent CVD, physicians recommend that patients quit smoking; eat a diet in which approximately 30% of the calories come from fat, choosing polyunsaturated fats and avoiding saturated fats and trans fats; reduce high blood pressure; increase physical activity; and maintain their weight within normal limits [53].

Hydroxymethylglutaryl-coenzyme A reductase inhibitors (statins) lower cholesterol synthesis. Statins represent the cornerstone for the treatment of hypercholesterolemia and in the prevention of CVD, although muscle-related side effects have strongly limited patients’ adherence and compliance [53]. The evidence in support of muscle pain caused by statins is in some cases equivocal and not particularly strong. The reported symptoms are difficult to quantify and rarely is it possible to establish a causal link between statins and muscle pain. In randomized controlled trials, statins have been well tolerated, and muscle pain–related side effects were similar to those caused by placebo. An exchange of statins may be beneficial, although all statins have been associated with muscle pain. In some patients, a reduction of dose is worth trying, especially in primary prevention [54]. Statins have been linked also to digestive problems, mental fuzziness, and glucose metabolism, and they may rarely cause liver damage. The influence of the diabetogenic action of statins is still unclear. Despite these observations, the CVD preventive benefit of statin treatment outweighs the CVD risk associated with the development of new diabetes [55]. There is good evidence that statins given late in life to people at risk for vascular disease do not prevent cognitive decline or dementia [56]. Statins can cause transient elevation of liver enzymes, which has led to the unnecessary cessation of these substances prematurely [57]. Coenzyme Q10 (CoQ10) is widely used as a dietary supplement, and one of its roles is to act as an antioxidant. Decreased levels have been shown in diseased myocardium and in Parkinson disease. Farnesyl pyrophosphate is a critical intermediate for CoQ10 synthesis, and blockage of this mechanism may be important in statin myopathy. Supplementation with CoQ10 has been reported to be beneficial in treating hypertension, statin myopathy, heart failure, and problems associated with chemotherapy; however, this use of CoQ10 as a supplement has not been confirmed in randomized controlled clinical trials [58].

In conclusion, recent analyses and randomized controlled trials have been published confirming that the cardiovascular benefits of statin therapy in patients for whom it is recommended by current guidelines greatly outweigh the risks of side effects [59]. The Cholesterol Treatment Trialists Collaboration meta-analysis showed that for each 1 mmol/L reduction in LDL, major vascular events (myocardial infarction, coronary artery disease death, or any stroke or coronary revascularization) were reduced by 22% and total mortality was reduced by 10% over 5 years [59].

### Extracting Categories From Raw Data

Our data set contains 1986 unique sentences about cholesterol and statins. Of the 1986 sentences, 1041 (52.42%) were labeled by medical experts as credible, 551 (27.74%) as neutral, and 394 (19.84%) as noncredible. We have reviewed the compliance of the assessments in the noncredible class with the annotation protocol. As a result, of the 394 noncredible annotations, 72 (18.3%) were discarded as noncompliant. The following are some examples of sentences erroneously annotated as noncredible:

“Why are they putting patient lives at risk?” Sentence is a question and should be labeled as neutral.

“Researchers chose 30 studies in total to analyze.” Sentence does not contain any medical terms and should be labeled as neutral.

“They [statins] work by blocking an enzyme called HMG-CoA reductase, which makes your body much slower at synthesizing cholesterol.” Sentence contains factually true statement and should be labeled as credible.

Finally, of the 1986 sentences, we identified 322 (16.21%) as noncredible. We extracted 18 claim categories, which represented 61.5% (198/322) of all noncredible sentences. The process of claim category extraction involved the following steps:

1. The annotator examined all the sentences from the noncredible class one by one.
2. If a sentence matched an already existing category, it was assigned to that category; otherwise, a new category was created.
3. After processing all the sentences, categories with only 1 sentence were merged into a Miscellaneous category that contained the remaining 29.5% (95/322) of the noncredible sentences.

We also compared the compliance of the extracted claim categories with current medical guidelines and knowledge. The category counts are presented in Table 4, and these categories are listed and explained in Table 5

**Table 4:** The number of occurrences of a particular claim category within the *cholesterol* and *statins* subset of sentences.

Claim category	Number of occurrences	Is related claim factually incorrect?	Is category based on the content or on the form?
Miscellaneous	95	N/A^a^	Form
(stat) Side effects	43	Yes	Content
(chol) Not an indicator of CVD^b^ risk	25	Yes	Content
Diet as good as drugs	22	Yes	Form
(chol) Too low is harmful	18	Yes	Content
Lifestyle changes are enough	15	Yes	Content
Big pharma	14	Yes	Content
Inflammation theory	14	Yes	Content
(stat) Cause diabetes	13	Yes	Content
(stat) Not needed	10	Yes	Content
(chol) Makes cells and protects nerves	8	No	Content
(stat) Not effective	7	Yes	Content
(stat) Prescription based solely on (chol) level	7	Yes	Content
Detailed data	7	N/A	Form
(stat) Cause cognitive impairment	6	Yes	Content
(stat) Not studied enough	6	Yes	Content
High HDL^c^ neutralizes high LDL^d^	6	No	Content
Harmful CoQ10^e^ loss	4	Yes	Content
(chol) Consumption not an issue	3	Yes	Content
Lifestyle versus statins	2	Yes	Content
No liver function monitoring	2	Yes	Content

^a^N/A: not applicable.

^b^CVD: cardiovascular disease.

^c^HDL: high-density lipoprotein.

^d^LDL: low-density lipoprotein.

^e^CoQ10: Coenzyme Q10.

**Table 5:** Claim category and explanations of claim categories extracted manually from all noncredible sentences from the *cholesterol* and *statins* topic.

Claim category	Claim explanation
(stat) Side effects	Statins’ side effects outweigh the benefits
(chol) Not an indicator of CVD^a^ risk	Total cholesterol is not an indicator of CVD
Diet as good as drugs	Aggregation of different dietary interventions to lower cholesterol, triglycerides, or sugars
(chol) Too low is harmful	Too low cholesterol level is harmful
Lifestyle changes are enough	People can lower cholesterol level just by developing good habits and eating a proper diet
Big pharma	People (eg, physicians and pharmaceutical company workers) make considerable profit through prescribing statins
Inflammation theory	It is inflammation that causes CVD, not excessive cholesterol level; cholesterol is an effect, not a cause
(stat) Cause diabetes	Statins increase the risk of diabetes
(stat) Not needed	Statins are given to healthy people who do not need them
(chol) Makes cells and protects nerves	Cholesterol produces hormones that make body cells and protect nerves
(stat) Not effective	Statins do not fulfill their role in reducing the risk of CVD
(stat) Prescription based solely on (chol) level	Statin prescription is based solely on total cholesterol level
Detailed data	Sentences contain detailed data, for example, “LDL^b^ cholesterol level should not exceed 200 md/dL”
(stat) Cause cognitive impairment	Statin consumption causes different forms of cognitive impairment (including memory loss and slow information processing)
(stat) Not studied enough	Statins’ effectiveness is not studied enough
High HDL^c^ neutralizes high LDL	HDL is a so-called good cholesterol, whereas LDL is a so-called bad cholesterol; high levels of the former neutralize negative consequences of high levels of the latter
Harmful CoQ10^d^ loss	Statin-related CoQ10 loss is harmful
(chol) Consumption not an issue	People should not worry about cholesterol consumption
Lifestyle versus statins	Lifestyle changes are more effective ways to prevent CVDs than statin consumption
No liver function monitoring	Monitoring of liver function tests is no longer recommended in patients on statin therapy
Miscellaneous	None of the above

^a^CVD: cardiovascular disease.

^b^LDL: low-density lipoprotein.

^c^HDL: high-density lipoprotein.

^d^CoQ10: Coenzyme Q10.

Of the 322 noncredible sentences, 198 (61.5%) fall into specific claim categories. Most of the categories have at least 6 examples that spread across different documents. We have designated categories with only 2 or 3 occurrences as separate because the entire noncredible class is relatively small and finding even a few similar sentences may indicate that the claim is being duplicated on the web.

Of the 95 sentences that did not fall into any claim category, we identified 9 (9%) that bear the hallmarks of a conspiracy theory, 7 (7%) containing reasoning based on anecdotal evidence, and 9 (9%) containing misleading statistical reporting:

- Conspiracy theory (referring to groups of interests such as prostatin vs antistatin researchers): “Ironically, prostatin researchers themselves are the ones who are guilty of cherry-picking.”
- Anecdotal evidence: “What’s worse, my doctor has never asked if I smoke cigarettes, exercise regularly, or eat a healthy diet.”
- Misleading statistical evidence: “OK, maybe the benefits of taking a statin are small, but many smart doctors say a reduction of five-tenths or six-tenths of 1% is worthwhile.”

As part of qualitative analysis, we compared 2 sets of clusters: automatically created versus manually created. We were able to select sentences that contain similar words and statements but differ in the narrative details that skewed the experts’ judgments. We have identified 4 types of false and misleading narratives that occur frequently in the noncredible class. These narratives are as follows:

1. Slippery slope: The sentence is factually true, but the consequences of the presented fact are exaggerated. Example:

Hence, while the drug might synergise with a statin to prevent a non-fatal (or minor) heart attack, it seems to increase the risk of some other equally life-threatening pathology, resulting in death.

Cholesterol also helps in the formation of your memories and is vital for neurological function.

2. Hedging: The sentence is factually incorrect, but there is a part of it that softens the overtone of the presented statement. Example:

However, cholesterol content should be less of a concern than fat content.

CRED

Coenzyme Q10 supplements may help prevent statin side effects in some people, though more studies are needed to determine any benefits of taking it.

CRED

The FDA warns on statin labels that some people have developed memory loss or confusion while taking statins.

CRED

3. Suggested negative consequences: The sentence is mostly factually true, but given the context of the expert’s experience, there is a risk that the presented information may lead the patient to act contrary to current medical guidelines. Examples:

For starters, statin drugs deplete your body of coenzyme Q10 (CoQ10), which is beneficial to heart health and muscle function.

Cholesterol is a waxy, fatty steroid that your body needs for things like: cell production.

4. Twisting words: the presence of a single word changes the overtone of the sentence. Examples:

*Statins may slightly increase the risk for Type 2 diabetes, a condition that can lead to heart disease or stroke.* [CRED]

*For example, it may be enough to eat a nutritious diet, exercise regularly, and avoid smoking tobacco products.* [NONCRED]

versus

Eating a healthy diet and doing regular exercise can help lower the level of cholesterol in your blood.

CRED

## Discussion

### Principal Findings

The results of our experiments show that applying the active annotation paradigm for credibility assessment in the medical domain produces measurable gains in terms of the use of medical experts’ time. Active annotation allows us to raise the number of noncredible statements annotated by medical experts by 30% on average, within a fixed time and monetary budget. Annotation of medical information cannot be crowdsourced because it requires the deep and broad domain knowledge of medical experts and their time is expensive. We regard the problem of prohibitively expensive annotation costs as the main obstacle to the broad use of machine learning models in the evaluation of the credibility of web-based medical resources. Our proposal is a step toward a significant lowering of these costs.

However, there is still room for improvement. Our qualitative analysis shows that most of the noncredible sentences can be classified into a limited number of categories. The subset of approximately 200 noncredible sentences from the *cholesterol and statins* subdomain can be divided into 18 categories, each representing approximately one false statement. These 18 categories fall into 61.5% (198/322) of the total number of all sentences labeled in full accordance with the annotation protocol. This indicates the importance of precise semantic clustering. More accurate clustering helps to detect noncredible sentences faster. It also enables the tagging of clusters with topic-related labels by nonexperts for later reviewing by medical experts and, as a result, the even more useful sentence ranking. In other words, it might be possible to use crowdsourcing to some extent during preprocessing and include an expert in the loop in the main annotation pipeline, further reducing the annotation costs.

Another conclusion that we drew from the qualitative analysis concerns the precision of the semantic similarity measure based on sentence embeddings. The method captures well the overall theme of the sentence but often misses the stance of the presented claim. This error is understandable because the stance in the medical domain is often expressed through subtle sentence modifications, as listed in the *Results* section. Sentence embeddings also struggle with finding a good representation of the form of the sentence—whether it is a supposition, a question, or a statement. Recognition of the form of the sentence can improve the accuracy of classification of neutral sentences that do not require medical expert annotation.

Finally, the qualitative analysis has revealed 4 distinct narratives present in noncredible sentences. Although our analysis was limited to the topic of cholesterol and statins, we feel that these narratives are more general in nature and may apply broadly to false medical information on other topics. If this hypothesis is confirmed, it may be possible to develop machine learning models for these narratives (eg, a model searching for instances of hedging expressions or words capable of twisting the stance of the sentence). Tagging these narratives during credibility annotation may not only increase the precision of sentence classifiers built upon such data sets, but, most importantly, also help disambiguate experts’ labeling process.

### Conclusions and Future Work

With the web quickly becoming one of the primary sources of the first medical information for the general public [60], the ability to distinguish between credible and noncredible information is indispensable. Financial interests of the alternative medicine community, combined with the rising distrust of the medical establishment, produce voluminous corpora of medical information of questionable quality. Of note, too many people fall prey to medical misinformation because it becomes increasingly harder to tell credible content from harmful deceit.

A possible solution to the problem of medical information source credibility is external certification. In our experiments, we correlated medical experts’ labels with HON labels. The certification certainly works because only 18% (240/1333) of the sentences originating from HON-certified websites were classified by our experts as noncredible. However, obtaining the certificate is not simple, the certification process is long, and the entire framework does not scale well. This scalability problem demonstrates the bottleneck of any approach used for checking the credibility of medical content—the availability and time of medical professionals who need to be involved in the evaluation. In our work, we have taken the approach of optimizing the use of the time spent by experts on credibility evaluation of medical web content. The main goal of our future work will be the improvement and extension of this approach using active annotation and active learning methods.

In contrast, an ambitious goal would be to replace medical experts’ evaluations with an automated credibility evaluation system. Such a system would use advanced natural language processing and machine classification algorithms. The results of our research demonstrate the challenges that would need to be overcome to make this possible.

The computational linguistic community is currently divided into 2 opposing camps: those who attribute *understanding of meaning* to language models and those who do not [61]. Despite the recent successes of modern language models such as Generative Pre-trained Transformer 3, the evidence seems to support a more cautious position. Indeed, a language model trained only on the form (raw text) cannot capture the true meaning of the text. The meaning, in this context, should be understood as the relationship between the linguistic form and the communicative intent of the speaker.

Our case goes beyond the learning of the meaning of sentences. As we have shown in this paper, there is an additional layer of complexity introduced by the notion of credibility of a statement to a user. Many machine learning solutions focus on the identification of factual flaws when addressing misinformation. However, fact-checking is not enough in the medical information domain. Often one encounters fake news and disinformation woven around factually true statements. We have seen time and time again medical experts using contextual information when assigning labels denoting sentence credibility. Most often they would take into account the most probable course of action taken by a patient who consumes medical information. Because of this mechanics of annotation, the relationship between sentence credibility and sentence truthfulness becomes ambiguous, further complicating the shape of the decision boundary between credible and noncredible medical statements.

This observation leads us to an important conclusion about the design of information-processing pipelines for medical content credibility evaluation. The first step is the compilation of large, high-quality data sets for machine learning model training. The active annotation approach presented in this paper allows doubling the number of sentences annotated by medical experts per cost unit (time or monetary). This, in turn, results in larger and more comprehensive training data sets. As a side effect, active annotation produces topical clusters of sentences, which can be used in 2 ways: (1) by allowing nonexpert annotators (whose time is far less expensive) to preprocess large batches of sentences to be reviewed by medical experts and (2) by reducing the cognitive stress of expert annotators due to the removal of context switching.

These 2 effects combined can further enhance the annotation process and increase the volume of annotated data. We also plan to extend the scope of the data set by covering more topics and providing more annotations.

The second step toward the support of medical content credibility evaluation would be the investigation of statistical models’ efficacy for automatic classification of medical sentences as either credible or noncredible. Having an accurate classifier of medical sentence credibility, we might develop machine-assisted methods for finding consensus among human annotators, for example, by correlating human annotations with the confidence scores of the classifier. Finally, we would like to pursue active annotation in the light of 2 frameworks. Bayesian reasoning provides a set of tools for modeling individual annotators’ beliefs about annotated data. Expectation maximization, in contrast, allows finding the best approximations (or maximum a posteriori estimates) of the unknown point credibility scores from empirical data. We see several possibilities of including the active annotation step in the iterative processes of Bayesian inference or expectation maximization.

## Appendix

Multimedia Appendix 1 Queries used to retrieve articles.

Multimedia Appendix 2 List of article URLs.

## Abbreviations

BERT: Bidirectional Encoder Representations from Transformers
CoQ10: Coenzyme Q10
CVD: cardiovascular disease
HON: Health on the Net
LDL: low-density lipoprotein
sBERT: sentence–Bidirectional Encoder Representations from Transformers
