# Supplementary material for: Active Annotation in Evaluating the Credibility of Web-Based Medical Information: Guidelines for Creating Training Data Sets for Machine Learning
Source: JMIR Med Inform. 2021 Nov 26;9(11):e26065. doi: 10.2196/26065 (PMC8665397; doi:10.2196/26065)
Supplement: Multimedia Appendix 1 [file medinform_v9i11e26065_app1.docx]

# Appendix 1

queries used to retrieve articles

| Natural birth vs. C-section | *“cesarean section natural childbirth”* |
| --- | --- |
| Steroids for kids | *”steroids cough treatment child”,*  *”child steroid dangers”* |
| Antibiotics for kids | *”antibiotics children”,*  *”don’t use antibiotics for kids”* |
| Autism & diet | *”diet autism”* |
| Food allergy testing | *”igg panel testing”,*  *”food allergy testing”,*  *”lactose breastfeeding”,*  *”elimination diet pregnancy”* |
| Antioxidants | *”antioxidants must”,*  *”choosing the best antioxidant supplements”* |
| Heart supplements | *”supplements for heart”* |
| Psychiatry | *”trust psychiatry”,*  *”antidepressants stopped working”,*  *”antidepressants addiction”,*  *”psychiatric drugs thinking”,*  *”anxiety alcohol”,*  *”psychotropic drugs effectiveness”,*  *”electroconvulsive therapy”* |
| Cholesterol & statins | *”cholesterol myth”,*  *”cardiologists against statins”,*  *”cholesterol avoid”,*  *”cholesterol dangerous”* |
| Vaccines | *”vaccines too many”,*  *”danger of multiple vaccines”,*  *“vaccines safe”* |
| Aspirin & pregnancy | *”aspirin pregnancy”* |
| SSRI during pregnancy | *”ssri mothers babies”* |
