# Supplementary material for: Active Annotation in Evaluating the Credibility of Web-Based Medical Information: Guidelines for Creating Training Data Sets for Machine Learning
Source: JMIR Med Inform. 2021 Nov 26;9(11):e26065. doi: 10.2196/26065 (PMC8665397; doi:10.2196/26065)
Supplement: Multimedia Appendix 2 [file medinform_v9i11e26065_app2.docx]

# Appendix 2

list of article URLs

| **url** | **is HON?** |
| --- | --- |
| <https://www.theguardian.com/society/2015/may/12/psychiatric-drugs-more-harm-than-good-expert> | no |
| <https://www.drugs.com/article/statins-benefits-and-risks.html> | no |
| <https://www.quora.com/Can-we-give-kids-antibiotics-3-times-a-month> | no |
| <https://www.infantrisk.com/antidepressant-use-during-pregnancy-and-breastfeeding> | no |
| <https://www.bda.uk.com/resource/food-allergy-intolerance-testing.html> | no |
| <https://www.livescience.com/34712-ldl-cholesterol-buildup-causes-heart-attack.html> | no |
| <https://www.intolerancelab.co.uk/testing/?gclid=Cj0KCQiAz53vBRCpARIsAPPsz8XcWyYu79jrJU0TP474JGyL0kCyOmSPeNR-A1a3pdnwzhgPTWuL3kcaAmbwEALw_wcB> | no |
| <https://www.newscientist.com/article/dn27929-stop-glossing-over-the-risks-of-natural-birth-to-cut-caesareans/> | no |
| <https://hipoalergiczni.pl/sterydy-dla-dzieci-horror/> | no |
| <https://www.verywellfamily.com/aspirin-and-risk-of-miscarriage-2371752> | no |
| <https://www.tommys.org/pregnancy-information/labour-birth/caesarean-section/c-section-benefits-and-risks> | no |
| <https://www.quora.com/Is-it-true-that-about-a-third-of-antibiotics-given-to-children-are-not-needed> | no |
| <https://www.healthline.com/health/food-allergy-testing#takeaway> | yes |
| <https://utswmed.org/medblog/statins-debate/> | no |
| <https://kidshealth.org/en/parents/allergy-tests.html> | yes |
| <https://www.healthline.com/health-news/cholesterol-levels-are-decreasing-better-heart-health#Room-for-improvementhttps> | yes |
| <https://www.quora.com/Why-are-gluten-free-diets-recommended-for-people-with-autism> | no |
| <https://www.nytimes.com/2018/04/16/well/weighing-the-pros-and-cons-of-statins.html> | no |
| <https://www.uspharmacist.com/article/ssri-use-by-mothers-leads-to-more-nicu-admissions-morbidity-in-newborns> | no |
| <https://www.cchr.org/cchr-reports/important-notice-for-the-reader.html> | no |
| <https://howtoadult.com/treat-diarrhea-children-2277802.html> | no |
| <https://www.quora.com/What-are-some-psychiatric-lies> | no |
| <https://www.mdedge.com/cardiology/article/157522/preventive-care/fight-statin-phobia-hard-facts> | no |
| <https://www.statnews.com/2015/12/01/cesarean-section-childbirth/> | no |
| <https://naturopathicearth.com/2019/01/18/disturbing-facts-about-psychotropic-drugs/> | no |
| <https://www.globenewswire.com/> | no |
| <https://www.sandiegouniontribune.com/news/health/sd-me-statins-heart-20161116-story.html> | no |
| <https://www.bbc.com/news/health-49795257> | no |
| <https://www.choosingwisely.org/patient-resources/antibiotics-for-ear-infections-in-children/> | no |
| <https://www.scarymommy.com/dairy-free-breastfeeding-mother/> | no |
| <https://parenting.pl/dieta-dla-dzieci-z-autyzmem> | no |
| <https://www.webmd.com/baby/news/20170628/aspirin-a-day-to-keep-pregnancy-problems-away#1> | yes |
| Not valid | no |
| <https://www.cchr.org/quick-facts/psychiatric-drugs-side-effects.html> | no |
| <https://foodforthebrain.org/condition/autism/> | no |
| <https://www.practo.com/healthfeed/7-reasons-why-vaccine-should-not-be-given-30705/post> | no |
| <https://www.successfulbreastfeeding.org/blog/dairy-intolerance-or-lactose-overload> | no |
| <https://www.theatlantic.com/health/archive/2013/05/the-real-problems-with-psychiatry/275371/> | no |
| <https://www.childrensmn.org/educationmaterials/childrensmn/article/15864/gluten-free-casein-free-diet-for-autism-spectrum-disorder/> | no |
| <https://www.medicalnewstoday.com/articles/327060#3> | no |
| <https://www.heartuk.org.uk/low-cholesterol-foods/foods-that-contain-cholesterol> | no |
| <https://www.the-scientist.com/the-nutshell/ssri-consumption-during-pregnancy-linked-to-changes-in-babies-brains-30114> | no |
| <https://www.drsinatra.com/the-great-cholesterol-myth> | no |
| <https://autismawarenesscentre.com/my-experience-with-the-gfcf-diet/> | no |
| <https://mamotoja.pl/aspiryna-ulatwia-zaplodnienie-i-zmniejsza-ryzyko-poronienia,przygotowania-do-ciazy-artykul,18666,r1p1.html> | no |
| <https://www.mamazone.pl/artykuly/ciaza-i-porod/ciaza/zdrowie/aspiryna-w-ciazy/> | no |
| <https://iancommunity.org/cs/what_do_we_know/special_diets> | no |
| <https://vaccinechoicecanada.com/health-risks/autism/the-impact-of-administering-several-vaccines-at-one-time/> | no |
| <https://edition.cnn.com/2019/01/08/health/cardiologist-statin-cholesterol-mission/index.html> | no |
| <https://ectjustice.com/no-one-should-be-given-shock-treatment/> | no |
| <https://allthenourishingthings.com/5-natural-remedies-for-ear-infections-no-antibiotics/> | no |
| <https://www.healthline.com/health/alcohol-and-anxiety> | yes |
| <http://www.hadassah-med.com/> | no |
| <https://iythealth.com/side-effects-thimerosal-vaccines/> | no |
| <https://www.manipalcigna.com/health-and-wellness/caesarean-section-vs-normal-birth> | no |
| <https://www.verywellhealth.com/do-i-really-need-to-worry-about-my-high-cholesterol-698283> | yes |
| <https://newsnetwork.mayoclinic.org/discussion/home-remedies-when-to-use-and-not-use-antibiotics-2/> | no |
| <https://consent.yahoo.com/v2/collectConsent?sessionId=3_cc-session_19c69979-3286-4f40-9b3e-8793b121cab3> | no |
| <https://www.quora.com/Should-I-trust-my-psychiatrist> | no |
| <https://kidshealth.org/en/parents/fact-myth-immunizations.html> | yes |
| <https://www.nhs.uk/conditions/food-intolerance/> | no |
| <https://articles.mercola.com/sites/articles/archive/2019/09/02/higher-cholesterol-is-associated-with-longer-life.aspx#_edn19> | no |
| <https://www.change.org/p/ban-electroshock-ect-device-being-used-on-children-the-elderly-and-vulnerable-patients> | no |
| <https://www.reuters.com/article/us-health-heart-supplements/few-supplements-have-proven-heart-benefits-idUSKCN1U42MS> | no |
| <https://childrenshealthdefense.org/too-many-sick-children/autism/> | no |
| <https://www.parentcircle.com/article/10-reasons-why-one-should-opt-for-natural-over-caesarean-childbirth/> | no |
| <https://www.mayoclinic.org/diseases-conditions/food-allergy/diagnosis-treatment/drc-20355101> | yes |
| <https://time.com/5231462/antidepressants-during-pregnancy-fetal-brain-changes/> | no |
| <https://www.stuff.co.nz/national/health/101759156/antidepressants-dont-work-and-mental-health-system-is-failing-experts-say> | no |
| <https://kidshealth.org/en/parents/c-sections.html> | yes |
| <https://www.cardiovascularbusiness.com/topics/lipids-metabolic/most-supplements-do-little-protect-heart-health> | no |
| <https://www.parents.com/pregnancy/my-body/is-it-safe/which-medications-are-safe-during-pregnancy/> | no |
| <https://www.gosh.nhs.uk/conditions-and-treatments/medicines-information/short-term-steroid-treatment> | no |
| <https://freepsychiatricnews.com/top-five-reasons-not-to-trust-psychiatrists-and-pharmaceutical-companies/> | no |
| <https://top10supps.com/best-antioxidant-supplements/> | no |
| <https://paleolf.us/products> | no |
| <https://www.healthline.com/health-news/debate-over-vaccine-safety> | yes |
| <https://www.theepochtimes.com/countering-false-vaccine-safety-claims_2387621.html> | no |
| <https://www.webmd.com/baby/news/20140407/aspirin-advised-for-women-at-high-risk-for-pregnancy-complication#1> | yes |
| <https://www.drstevesilvestro.com/cough-croup-and-wheezing-in-kids> | no |
| <https://www.znana-polozna.pl/artykuly/zespol-reyea-czyli-o-kwasie-acetylosalicylowym-w-ciazy> | no |
| <https://www.verywellhealth.com/prednisone-medication-information-2633522> | yes |
| <https://www.spectrumnews.org/news/analysis-finds-little-evidence-to-support-dietary-interventions-for-autism/> | no |
| <https://www.nature.com/articles/d41586-019-02807-x> | no |
| <https://www.smithsonianmag.com/science-nature/babies-born-c-section-found-have-different-gut-microbes-vaginally-delivered-infants-180973174/> | no |
| <https://www.medicalnewstoday.com/articles/317332#cooking-techniques-and-tips> | yes |
| <https://www.babycenter.com/pregnancy/health-and-safety/is-it-safe-to-take-aspirin-during-pregnancy_1357403> | no |
| <https://www.scmp.com/lifestyle/health-beauty/article/2126118/how-stop-your-baby-having-food-allergies-eat-right-during> | no |
| <https://thefitbay.com/best-antioxidants-reviews/> | no |
| <https://www.whattoexpect.com/family/childrens-health-and-safety/overusing-antibiotics-in-children/> | no |
| <https://www.scientificamerican.com/article/electroconvulsive-therapy-a-history-of-controversy-but-also-of-help/> | no |
| <https://wyborcza.pl/TylkoZdrowie/1,137474,17315059,Nie_mieszaj____ciazy_z_aspiryna.html?disableRedirects=true> | no |
| <https://www.med.unc.edu/psych/patient-care/adult/outpatient/electroconvulsive-therapy-ect-service/> | no |
| <https://www.drsinatra.com/why-your-cholesterol-test-can-lie> | no |
| <https://answers.yahoo.com/question/index?qid=20110628003844AAASLjz&page=2&guccounter=1&guce_referrer=aHR0cHM6Ly9tZWRjcmVkaWJpbGl0eXN1cnZleXMuaGVyb2t1YXBwLmNvbS9hZG1pbi9pbmRleA&guce_referrer_sig=AQAAAEvMlGLwYtiGb9M-VfBTJYO4ryZeEyci-dnMYFTab5rySoBlcErg5eAilunmsxlLHOSHbHFPunrh6gkOvjPgBoE6yBHoHajWZm8DNj-eTr_y7qM-PDhKLl5cUTMl9ScVnYtPKdGSQFOYqJUt7vX-nh7cPMf7WdBPUH8lG-62Ws7u> | no |
| <https://www.choosingwisely.org/patient-resources/dietary-supplements-to-prevent-heart-disease-or-cancer/> | no |
| <https://www.webmd.com/children/news/20110901/study-kids-are-getting-too-many-antibiotics#1> | no |
| <https://www.neurologyadvisor.com/conference-highlights/aaic-2018/some-statins-may-be-associated-with-cognition-memory-deficits/> | no |
| <https://www.abc.net.au/news/health/2017-04-21/can-you-have-too-many-antioxidants/8457336> | no |
| <https://www.mayoclinic.org/diseases-conditions/high-blood-cholesterol/in-depth/statin-side-effects/art-20046013> | yes |
| <https://www.nhs.uk/live-well/healthy-body/lower-your-cholesterol/> | no |
| <https://www.naturalnews.com/042769_antibiotics_healthy_gut_flora_immune_system.html> | no |
| <https://www.peacehealth.org/healthy-you/8-heart-health-supplements-take-and-one-avoid> | no |
| <https://www.akademiadietetyki.pl/dietetyka/dieta-recepta-na-autyzm/> | no |
| <https://www.top10homeremedies.com/news-facts/why-antibiotics-are-harmful-for-your-health.html> | no |
| <https://www.medpagetoday.com/cardiology/cardiobrief/66973> | no |
| <https://pubmed.ncbi.nlm.nih.gov/1285867/> | no |
| <https://www.quora.com/Why-shouldnt-I-use-alcohol-2-3-drinks-per-day-to-help-with-my-anxiety-disorder> | no |
| <https://community.babycenter.com/post/a24262301/pregnant_and_allergy_elimination_diet> | no |
| <https://www.cbc.ca/news/canada/montreal/quebec-electroshock-therapy-protest-1.3572139> | no |
| <http://vitals.nbcnews.com/_news/2013/01/16/16546291-crowded-vaccine-schedule-for-babies-safe-study-finds?lite> | no |
| <https://www.collective-evolution.com/category/health/> | no |
| <http://swiatlekarza.pl/sterydy-kontroluja-objawy-astmy/> | no |
| <https://www.babyboom.pl/maluszek/zdrowie/dieta-dziecka-autystycznego> | no |
| <https://www.express.co.uk/life-style/health/1162761/best-supplements-heart-magnesium-cinnamon-omega-3-fatty-acid-folic-acid-mediterranean-diet> | no |
| <https://ravimid.files.wordpress.com/2015/02/long-lasting-negative-effects-090314.pdf> | no |
| <https://www.newscientist.com/article/2199880-statins-may-not-lower-cholesterol-enough-in-half-those-who-take-them/> | no |
| <https://familydoctor.org/antioxidants-what-you-need-to-know/> | no |
| <https://www.healthline.com/health/high-cholesterol/why-statin-drugs-may-be-bad-for-you> | yes |
| <https://hub.jhu.edu/2017/01/11/vaccines-autism-public-health-expert/> | no |
| <https://www.hopkinsmedicine.org/health/wellness-and-prevention/antidepressants-and-pregnancy-tips-from-an-expert> | no |
| <https://www.nps.org.au/australian-prescriber/articles/the-management-of-croup> | no |
| <https://youqueen.com/life/3-natural-antibiotics-for-kids/> | no |
| <https://www.healio.com/news/primary-care/cardiology/news> | no |
| <https://www.forbes.com/sites/matthewherper/2012/03/04/top-cardiologist-argues-we-should-dial-back-on-statins-because-of-diabetes-risk/> | no |
| <https://www.medicalnewstoday.com/articles/325692#5> | yes |
| <https://sciencebasedmedicine.org/igg-food-intolerance-tests-continue-to-mislead-consumers-into-unnecessary-dietary-restrictions/> | no |
| <https://www.autismspeaks.org/nutrition-and-autism> | no |
| <https://tasteforlife.com/supplements/vitamins-minerals/10-supplements-for-a-healthy-heart> | no |
| <https://translationalneurodegeneration.biomedcentral.com/articles/10.1186/2047-9158-3-16> | no |
| <https://www.thedailybeast.com/are-kids-getting-too-many-vaccines> | no |
| <https://www.everlywell.com/products/food-sensitivity/> | no |
| <https://www.publichealth.org/public-awareness/understanding-vaccines/vaccine-myths-debunked/> | no |
| <https://www.healthychildren.org/English/health-issues/conditions/allergies-asthma/Pages/Corticosteroids.aspx> | yes |
| <https://citizen.co.za/parenty/2095036/c-section-vs-natural-birth-which-is-better/> | no |
| <https://www.nhs.uk/live-well/healthy-body/lower-your-cholesterol/> | no |
| <https://www.medicalnewstoday.com/articles/325873> | yes |
| <https://polki.pl/rodzina/dziecko,czy-nalezy-bac-sie-sterydow,10352074,artykul.html> | no |
| <https://www.yorktest.com/products/complete-bundle/> | no |
| <https://blog.daveasprey.com/statin-side-effects/> | no |
| <http://autyzm.org.pl/strefa-rodzica/dieta-dziecka-z-autyzmem/> | no |
| <https://www.aaaai.org/conditions-and-treatments/library/allergy-library/IgG-food-test> | no |
| <https://www.whattoexpect.com/child-vaccinations/are-immunizations-safe.aspx> | no |
| <https://www.bmj.com/content/364/bmj.k5233> | no |
| <https://www.dermstore.com/blog/top_ten/antioxidants-in-skin-care/> | no |
| <https://www.healthline.com/nutrition/antioxidants-explained> | no |
| <https://www.europeanscientist.com/en/features/do-statins-really-work-who-benefits-who-has-the-power-to-cover-up-the-side-effects/> | no |
| <https://www.who.int/vaccine_safety/initiative/detection/immunization_misconceptions/en/index2.html> | yes |
| <https://www.babycentre.co.uk/x568978/is-it-safe-to-take-aspirin-during-pregnancy> | yes |
| <https://www.contemporarypediatrics.com/view/vaccines-are-safe-even-after-reactions> | no |
| <https://examine.com/nutrition/5-supplements-and-foods-for-a-stronger-heart/> | no |
| <https://takecareof.com/articles/guide-to-heart-health> | no |
| <https://www.nhs.uk/news/heart-and-lungs/study-says-theres-no-link-between-cholesterol-and-heart-disease/> | no |
| <https://www.nvic.org/vaccines-and-diseases/Diabetes/juvenilediabetes.aspx> | no |
| <https://www.incredibleegg.org/nutrition/articles/> | no |
| <https://www.health.com/condition/depression/10-myths-and-facts-about-shock-therapy> | no |
| <https://pubmed.ncbi.nlm.nih.gov/1545092/> | no |
| <https://www.heart.org/en/news/2018/12/10/safety-of-statins-emphasized-in-new-report> | no |
| <https://www.gdx.net/product/igg-food-antibodies-food-sensitivity-test-blood> | no |
| <https://www.naturesbest.co.uk/antioxidants/> | no |
| <https://academic.oup.com/qjmed/article/104/10/867/1591864> | no |
| <https://www.healthychildren.org/English/health-issues/conditions/chest-lungs/Pages/Croup-Treatment.aspx> | yes |
| <https://childrenshealthdefense.org/news/dtp-vaccine-increases-mortality-in-young-infants-5-to-10-fold-compared-to-unvaccinated-infants/> | no |
| <https://www.healthychildren.org/English/safety-prevention/immunizations/Pages/Vaccine-Safety-The-Facts.aspx> | yes |
| <https://www.livescience.com/45681-vaginal-birth-vs-c-section.html> | no |
| <https://www.testyourintolerance.com/product/choice-70-allergy-intolerance-test/> | no |
| <https://www.statnews.com/2019/04/03/statin-heart-disease-prevention-more-than-medicine/> | no |
| <https://www.hopkinsmedicine.org/health/conditions-and-diseases/high-cholesterol/3-myths-about-cholesterol-lowering-statin-drugs> | no |
| <https://www.eatright.org/health/allergies-and-intolerances/food-intolerances-and-sensitivities/are-food-sensitivity-tests-accurate> | no |
| <https://www.freedommag.org/issue/201411-held-back/reform/shock-treatment-truth-behind-electroshock-therapy.html> | no |
| <https://www.verywellfamily.com/low-dose-aspirin-and-miscarriages-2371795> | yes |
| <https://www.quora.com/What-psychiatric-illness-is-most-treatable-with-drugs-something-approaching-100-cure> | no |
| <https://parenting.pl/kwas-acetylosalicylowy-a-ciaza> | no |
| <https://iliveok.com/news/antibiotics-are-dangerous-children_90723i15820.html> | no |
| <https://blogs.scientificamerican.com/cross-check/are-psychiatric-medications-making-us-sicker/> | no |
| <https://a-fib.com/my-top-7-picks-natural-supplements-for-a-healthy-heart/> | no |
| <https://www.prnewswire.com/news-releases/cchr-ban-needed-on-torturous-electroconvulsive-therapy-300690928.html> | no |
| <https://www.seafoodsource.com/features/debunking-the-cholesterol-myth> | no |
| <https://healthydebate.ca/2017/01/topic/igg-tests-science> | no |
| <https://www.mcgill.ca/oss/article/controversial-science-food-health-supplements-toxicity/antioxidants-disappoint-again> | no |
| <https://www.escardio.org/Education/Practice-Tools/Talking-to-patients/cholesterol-and-statins> | no |
| <https://www.reuters.com/article/us-health-pregnancy-antidepressant-safet/antidepressants-in-pregnancy-tied-to-changes-in-babies-brains-idUSKBN1HG2WO> | no |
| <https://www.genre.com/knowledge/blog/the-drugs-dont-work-what-happens-when-antibiotics-fail-en.html> | no |
| <https://www.quora.com/Can-we-give-kids-antibiotics-3-times-a-month> | no |
| <https://www.madinamerica.com/2018/07/professional-delusion/> | no |
| <https://www.eurekalert.org/pub_releases/2004-02/mp-alb020204.php> | no |
| <https://www.rand.org/pubs/research_briefs/RB9799.html> | no |
| <https://globalnews.ca/news/4155705/c-section-birth-statistics-canada/> | no |
| <https://www.testyourintolerance.com/product/advanced/> | no |
| <https://newsnetwork.mayoclinic.org/discussion/mayo-clinic-minute-do-heart-supplements-work/> | no |
| <https://pulmccm.org/randomized-controlled-trials/oral-steroids-dont-improve-cough-due-colds-prescribed-anyway/> | no |
| <https://www.healthline.com/health/high-cholesterol#outlook> | no |
| <https://www.telegraph.co.uk/science/2016/06/12/high-cholesterol-does-not-cause-heart-disease-new-research-finds/> | no |
| <https://answers.yahoo.com/> | no |
| <https://www.today.com/health/are-vaccines-safe-answer-clear-yes-t106904> | no |
| <https://www.hopkinsmedicine.org/health/wellness-and-prevention/the-truth-about-heart-vitamins-and-supplements> | no |
| <https://healthpath.com/food-intolerance/are-food-allergy-and-food-intolerance-the-same/> | no |
| <https://www.healthline.com/health/vaccinations/opposition#common-reasons> | no |
| <https://drpaluchowska.pl/en/food-intolerance/> | no |
| <https://rmalab.com/medical-laboratory-tests/allergy/igg-sensitivity> | no |
| <https://www.amenclinics.com/blog/5-foods-can-make-autism-worse/> | no |
| <https://www.healthline.com/health/antidepressants-arent-addicting#1> | yes |
| <https://www.rehabspot.com/drugs/antidepressants/> | no |
| <https://www.addictioncenter.com/stimulants/antidepressants/> | yes |
| <https://sciencenordic.com/biology-denmark-depression/scientist-antidepressants-cause-addiction/1386012> | no |
| <https://medium.com/inspire-the-mind/are-antidepressants-addictive-yes-no-it-depends-7d0a3de1d638> | no |
| <https://www.health.harvard.edu/mind-and-mood/what-are-the-real-risks-of-antidepressants> | no |
| <https://www.theguardian.com/commentisfree/2019/may/30/antidepressant-withdrawal-symptoms-doctors-side-effects> | no |
| <http://www.deadlymedicines.dk/wp-content/uploads/2016/02/G%C3%B8tzsche-antidepressants-are-addictive-and-increase-risk-of-relapse-bmj.i574.full_.pdf> | no |
| <https://www.netdoctor.co.uk/healthy-living/advice/a25829/are-antidepressants-addictive/> | yes |
| <https://www.newscientist.com/article/mg23931980-100-nobody-can-agree-about-antidepressants-heres-what-you-need-to-know/> | yes |
| <https://americanaddictioncenters.org/withdrawal-timelines-treatments/anti-depressants> | no |
| <https://www.telegraph.co.uk/science/2019/09/19/common-antidepressant-barely-helps-improve-depression-symptoms/> | yes |
| <https://www.womenshealthmag.com/health/a27312284/reasons-your-antidepressant-isnt-working/> | no |
| <https://www.mayoclinic.org/diseases-conditions/depression/expert-answers/antidepressants/faq-20057938> | no |
| <https://theconversation.com/why-natural-depression-therapies-are-better-than-pills-106152> | no |
| <https://www.self.com/story/tolerance-to-antidepressant> | no |
| <https://www.wellandgood.com/antidepressant-tolerance-solutions/> | yes |
| <https://www.thesun.co.uk/news/9965578/britains-prescribed-antidepressant-barely-works/> | no |
| <https://www.verywellmind.com/what-to-do-if-your-antidepressant-has-stopped-working-1066864> | no |
| <https://www.bustle.com/articles/163511-what-to-do-if-your-antidepressants-stop-working> | no |
| <https://www.lipstickalley.com/threads/commonly-prescribed-antidepressant-sertraline-aka-zoloft-takes-up-to-12-weeks-to-ease-low-mood.2785075/> | no |
| <https://www.everydayhealth.com/depression/signs-your-antidepressant-isnt-working.aspx> | yes |
